# Materials Make a Better Life: Functional Metals, Metal Oxides, and Metal Complexes

**DOI:** 10.3390/ma16051899

**Published:** 2023-02-24

**Authors:** Piotr Piszczek, Aleksandra Radtke

**Affiliations:** Faculty of Chemistry, Nicolaus Copernicus University in Toruń, Gagarina 7, 87-100 Toruń, Poland

Materials based on metals, metal oxides, and metal complexes play an essential role in various areas of our lives. Their physicochemical and biological properties and their use depend on the type of metal, their structure, the morphology of their oxides, and, in the case of metal complexes, the type of stabilizing ligands. Industrial and nanotechnological developments have led to contamination of metals (metal nanoparticles) in our environment; their removal and recovery are essential. Moreover, in the case of metal nanoparticles, it is also important to know their toxic effects on our bodies and the environment. The presented Special Issue “Materials Make a Better Life: Functional Metals, Metal Oxides, and Metal Complexes” is a thematically diverse collection of eleven papers (two review articles and nine research articles). The scope of the discussed issues concerns the use of metal-based materials in medicine, the food industry, and environmental protection.

The synthesis and structure, as well as the photocatalytic and antimicrobial properties, of oxo-titanium(IV) complexes (TOCs) stabilized with acetylsalicylic acid ligands are discussed in articles [1,2]. The properties of the above compounds were studied using polymer (poly(ε-caprolactone))–TOC composite systems. It was found that the tested coating materials showed good activity towards photodecolorization of the methyl blue solution after irradiation with visible light. In addition, the antimicrobial activity of the tested materials during irradiation with visible light was proven. The results of the analysis of four new copper(II) complex compounds and their potential use as anticancer agents are presented in the article [3]. Derivatives of 6-bromo-2-(pyridin-3-yl)-3H-imidazo[4,5-b]pyridine copper(II) complexes have been found to be potential drug candidates against human lung adenocarcinoma. Moreover, it was noted that the compound mentioned above showed strong activity against the A549 cell line.

When studying issues related to the search for biomaterials and the possibility of their use in implantology, attention should be paid to the article [4]. The report discusses the results of research into cathodic deposition of hydroxyapatite onto the surface of Ti6Al4V substrates, which were previously modified by the production of nanoporous TiO_2_ coatings or TiO_2_ nanofibers or titanate coatings. It was proven that the presence of an intermediate titanium oxide coating significantly increases the adhesion between the hydroxyapatite (HA) layer and the Ti6Al4V substrate, thus providing a solution to the problem of delamination of the HA layer from the metallic substrate during extended implant use. An important issue related to producing materials based on titanium dioxide nanotubes is the scaling of their synthesis process [5]. Tests on titanium samples of various sizes have shown that, maintaining the same conditions as the anodic oxidation process, as their surface increases, the height of the formed nanotubes decreases and the distance between them increases, while the thickness of the nanotube walls is maintained. In addition, it was observed that changes in their outer diameter and the actual surface area did not affect the generated photocurrent. The composition of the resulting material also remained the same after increasing the size of the substrate. The results of the conducted research are important for using materials based on TiO_2_ nanotubes in medicine, photoelectrochemistry, and in the production of sensors and supercapacitors.

An interesting topic from the food industry’s point of view is the use of silver nanoparticles (AgNP) for detecting pesticides in fruits and vegetables [6]. The authors propose using a flexible substrate methylcellulose (MC) enriched with AgNP. Using the MC/AgNP system allowed the detection of thiram pesticide residues on the skins of cucumbers and tomatoes. Compared to existing technologies, the MC/Ag NP system could detect pesticides quickly without any other sample treatment. The use of iron nanoparticles (FeNP) in the food industry was addressed by the authors of article [7]. The high reactivity of FeNPs with oxygen both in the presence of moisture and in an anhydrous environment, and their ability to counteract the growth of microorganisms belonging to different groups (Gram-negative and Gram-positive bacteria, yeasts, and moulds), presents an interesting issue. It was concluded that FeNPs could be used in the production of food contact packaging as well as in the food itself. Smaller dimensions mean a larger external surface, which improves water absorption, aroma release, availability of bioactive substances, and the speed of the catalytic processes. However, certain limitations (e.g., concentrations of no more than 100 μg mL^−1^) related to FeNP use should be considered. The wide use of metal-based nanoparticles (MNPs) in various areas of our lives has led to the undertaking of research on the biosafety of their use. The article [8] reviewed works on the interaction between MNPs and Arabidopsis thaliana (a model plant). In order to determine the mechanisms that affect plant growth and development, attention was paid to various effects related to plant inter-metallization and phytotoxicity. The authors state that because studies have been performed at different levels of the plant’s response, i.e., subcellular, physiological, and biochemical levels, the interactions between MNPs and Arabidopsis should be designed in a way that combines the traditional toxicological research methods with histological techniques (transcriptomics, metabolomics, and proteomics) to provide more accurate and in-depth elucidation of the mechanisms of MNP-based phytotoxicity.

Intensive industrial development has polluted our environment with heavy metals. They are a significant problem because their ions are not biodegradable and accumulate in animal and human body tissues, as well as in water. One of the most common heavy metals in industrial discharge is zinc. A method detailing the removal of Zn(II) from aqueous synthetic solutions is proposed in article [9]. With this aim, laurel, canelo, and eucalyptus residues were used as the support of magnetic composites to be used as adsorbents in zinc removal. The optimal contact time was 3 h, and an adsorbent dosage of 7 g/L allowed the maximum removal of zinc, which was about 98.9, 98.8 and 97.6% for laurel, canelo, and eucalyptus magnetic composites, respectively. The issue of recovering metals (nickel and cobalt) from used lithium-ion batteries was dealt with by the authors of papers [10,11]. The advantages of lithium-ion batteries have led to them being the principal type of battery used in mobile phones, portable notebooks, and electric vehicles, among others. With the development of these batteries, their scrapping and disposal have become significant problems. Used lithium-ion batteries do not easily degrade and have a certain toxicity. Therefore, the recovery of metals from such kind of batteries is important from the point of view of environmental protection, as well as the reuse of recovered metals. Electrometallurgical technology has been employed in these research works. Reduction mechanisms of Ni(III) [10] and Co(III) [11] in molten Nal-CaCl_2_-LiMO_2_ salts (M = Ni and Co) lead to their separation and extraction from waste.

We want to thank all the authors for their submissions to this Special Issue. We also thank the reviewers for dedicating their time and helping to ensure the quality of the submitted papers. Moreover, we thank the editorial staff of *Materials* for their indispensable assistance.
